# Epidemiology of Merkel Cell Polyomavirus Infection and Merkel Cell Carcinoma

**DOI:** 10.3390/cancers14246176

**Published:** 2022-12-14

**Authors:** Steffi Silling, Alexander Kreuter, Thilo Gambichler, Thomas Meyer, Eggert Stockfleth, Ulrike Wieland

**Affiliations:** 1 Institute of Virology, National Reference Center for Papilloma- and Polyomaviruses, Faculty of Medicine, University Hospital Cologne, 50935 Cologne, Germany; 2 Department of Dermatology, Venereology and Allergology, HELIOS St. Elisabeth Hospital Oberhausen, University Witten/Herdecke, 58455 Witten, Germany; 3 Skin Cancer Center, Department of Dermatology, Ruhr-University Bochum, 44791 Bochum, Germany

**Keywords:** Merkel cell polyomavirus, MCPyV, Merkel cell carcinoma, MCC, epidemiology

## Abstract

**Simple Summary:**

Merkel cell polyomavirus (MCPyV) is a widespread virus that is present on human skin. Infection occurs early in life, and up to 90% of adults have antibodies against MCPyV. In older persons, a rare but aggressive cancer, Merkel cell carcinoma (MCC), can develop especially on sun-exposed skin at a fast-growing as a painless red nodule. Certain risk factors for MCC development have been identified, e.g., fair skin, male sex, older age (>70 years), and immunosuppression. In recent decades, the annual number of newly diagnosed MCC per 100.000 people has risen worldwide. In 80% of cases, MCPyV is the causative agent. Another entity of MCC is caused directly by DNA damage due to UV light. Both MCC entities have low survival and high recurrence rates unless diagnosed early. To raise awareness of this uncommon cancer, the epidemiology of MCPyV and MCC, as well as the characteristics of MCC, are reviewed in this article.

**Abstract:**

Merkel cell polyomavirus (MCPyV) is a ubiquitous virus replicating in human dermal fibroblasts. MCPyV DNA can be detected on healthy skin in 67–90% of various body sites, and intact virions are regularly shed from the skin. Infection occurs early in life, and seropositivity increases from 37 to 42% in 1- to 6-year-olds to 92% in adults. Merkel cell carcinoma (MCC) is a rare but very aggressive neuroendocrine tumor of the skin. It develops mainly on sun-exposed areas as a fast-growing, reddish nodule. Two MCC entities exist: about 80% of MCC are MCPyV-associated. Tumorigenesis is driven by viral integration into the host genome and MCPyV oncogene expression. In MCPyV-negative MCC, UV radiation causes extensive DNA damage leading to the deregulation of the cell cycle. In recent decades, MCC incidence rates have increased worldwide, e.g., in the United States, from 0.15 in 1986 to 0.7/100,000 in 2016. Risk factors for the development of MCC include male sex, older age (>75 years), fair skin, intense UV exposure, and immunosuppression. Projections suggest that due to aging populations, an increase in immunosuppressed patients, and enhanced UV exposure, MCC incidence rates will continue to rise. Early diagnosis and prompt treatment are crucial to reducing high MCC morbidity and mortality.

## 1. Introduction

Merkel cell polyomavirus (MCPyV), first discovered in Merkel cell carcinoma (MCC) in 2008 [1,2], is a small non-enveloped DNA tumor virus with a circular, double-stranded genome of approximately 5400 bp. MCPyV is one of fifteen currently known polyoma-viruses (PyV) found in humans [3]. Dermal fibroblasts are the cells preferentially infected by MCPyV in the human skin [4]. During the permissive cycle, the viral genome is maintained as an episome. It can be divided into two regions: the early region encodes the oncogenes large T (LT)-antigen and small T (sT)-antigens and has been shown to produce variably spliced transcripts, including a transcript that encodes a protein in an alternate reading frame from the large T antigen (called ALTO), and an autoregulatory miRNA. The late region codes for the structural proteins VP1 and VP2 [5]. MCPyV is the most frequent human polyomavirus (HPyV) detectable on healthy human skin [6]. Assembled virions are chronically shed from the skin, and MCPyV is considered to be part of the human skin virome [7,8].

To date, two phylogenetic groups of MCPyV have been described: one group comprises strains isolated from Caucasians, and the other group contains strains found in Japan that form a separate unique clade [7,8].

Merkel cell carcinoma (MCC) is a rare but very aggressive skin cancer with rising incidence rates. MCC tends to progress rapidly and metastasize early, which emphasizes the need for early detection, prompt confirmation of the diagnosis, and the immediate initiation of treatment [9]. This review summarizes recent data on the epidemiology of MCPyV infection and MCC from the up-to-date literature.

## 2. Methods

For this narrative review, a systematic search of the literature was carried out to identify all relevant articles using the National Institutes of Health PubMed search engine for articles in English. The following search terms were included in varying combinations: Merkel cell carcinoma, MCC, Merkel cell polyomavirus, MCPyV, epidemiology, incidence rate, and worldwide. The literature with incidence rates for specific countries/regions cited in recent publications was traced individually. Data from all studies reporting MCC incidence rates were included irrespective of the number of cases.

## 3. Epidemiology of MCPyV Infection

### 3.1. MCPyV Infection of the Skin

Primary infection with MCPyV seems to occur in the first year of life, either via the respiratory tract [10] or as a skin infection, presumably through direct skin contact with household members [11]. So far, the replication of MCPyV has been shown to be limited to fibroblasts from the dermis and potentially from the lung [4]. In skin smears from normal, lesion-free skin, MCPyV DNA can be detected in 49% to more than 80% of individuals, with no relevant differences regarding various body sites (forehead, back of the hand, trunk, upper and lower extremity). In one study, MCPyV-DNA was detected in 80% of swabs taken from different body sites of twenty-four healthy volunteers. MCPyV positivity rates were 92% (face), 81% (trunk), 64%, and 73% (upper and lower limb, respectively) [12]. Similarly, no differences were found between MCPyV-DNA baseline prevalence rates on the hand and forehead skin of 109 volunteers (67.0% and 67.9%, respectively) [6]. In a prospective study on MCPyV prevalence on the forehead, 49.4% of 239 healthy individuals were MCPyV DNA-positive [13]. In a case–control study, MCPyV DNA was also detected in eye-brow hairs of 300 controls, but to a lesser extent (37.3%) than in cutaneous swabs [14].

Both MCPyV prevalence rates and viral load increase with age. In individuals above 80 years of age, MCPyV DNA can be found on normal forehead skin in up to 95% [15]. Cutaneous long-term persistence of MCPyV for 10 years or more is associated with higher viral loads and can be found in about 40% of immunocompetent individuals. Risk factors for cutaneous MCPyV long-term persistence are coinfection with other human polyomaviruses and the male sex [6]. Along with beta and gamma human papillomaviruses and other HPyVs, such as HPyV6 and HPyV7, MCPyV is considered to be part of the human skin virome [6,16].

Compared to healthy individuals, cutaneous MCPyV prevalence has increased in HIV-positive patients. In forehead swabs of non-lesional skin, MCPyV DNA was found in 59.0% of 210 HIV-infected men versus 49.4% of 239 healthy male controls. MCPyV DNA loads were comparable in both groups, but individuals with poorly controlled HIV infection had significantly higher MCPyV DNA loads than those with well-controlled HIV infection. This could explain the elevated risk of MCPyV-associated MCC observed among HIV-positive individuals [13].

### 3.2. MCPyV Seroprevalence

**Figure 1:**
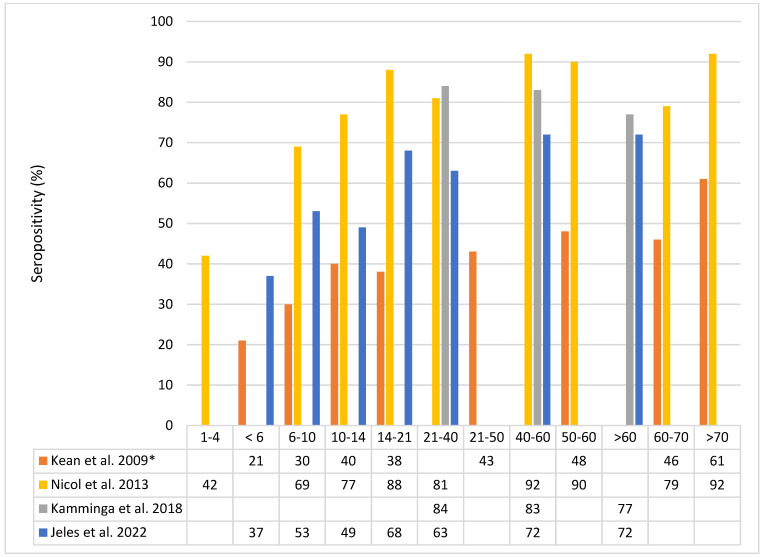
MCPyV seroprevalence in percent by age group in years. Data from Kean et al. 2009 [17] (*only antibodies against MCPyV isolate 339 are shown), Nicol et al 2013 [25], Kamminga et al. 2018 [23], and Jeles at al. 2022 [24].

In an early study from the United States (US), MCPyV seroprevalence in adults ranged between 25% (MCPyV strain 350) and 42% (major capsid protein VP1 of MCPyV strain 339) [11]. In more recent studies, higher MCPyV (VP1) seroprevalence rates were found: 82% in blood donors from the Netherlands [17], 69% in adults from Hungary [18], and up to 96% in Italian persons aged 70–79 years [19] (Figure 1).

Infection with MCPyV occurs early in life, and the prevalence of MCPyV-VP1-IgG in small children up to six years old is 37–42% and increases to 40–88% in teenagers and young adults up to 21 years of age (Figure 1). In healthy adults, the age-specific MCPyV seroprevalence rates that are reported for different countries vary, possibly due to the use of different serological assays, and range from 63 to 84% (21–40 years), 72 to 92% (40–60 years), and 72 to 92% in the over 60 years old [17,18,19].

High seroprevalence rates in people above the age of 70 years might be explained by the assumption that MCPyV is able to establish a reservoir of latently infected cells. Such latent infections might be resistant to clearance by neutralizing antibodies and, thus, could serve as a durable source of immunogenic virions [20]. Additionally, there are hints for viral clearance, as only 39.0% of healthy volunteers showed MCPyV long-term persistence (>9 years) on the forehead compared to 67.9% and 59.6% of people who were MCPyV-DNA-positive at the baseline or had short-term persistence, respectively [6].

In healthy individuals, there is a positive correlation between MCPyV virion-specific antibody titers and the detection of viral DNA in swabs from various anatomical skin sites [21]. Faust et al. found that 97% of MCPyV DNA-positive patients also had antibodies against MCPyV-VP1, and high titers correlated with high MCPyV DNA loads [22]. This argues for the skin is the primary site of chronic MCPyV infection and suggests that the magnitude of a person’s MCPyV-specific sero-responsiveness reflects the cumulative MCPyV DNA load of wide skin areas. An uncontrolled chronic MCPyV infection might be a risk factor for the development of MCC [21].

Although past exposure to MCPyV is common among adults, MCC patients with MCPyV-positive tumors have a markedly elevated MCPyV-VP1-IgG response (about 60-fold higher) compared with the control patients [21]. In another study, only four percent of controls showed neutralizing antibody titers above the mean value of MCPyV-positive MCC patients. These MCPyV VP1 capsid antibodies seem to be induced by an immunogenic MCPyV infection rather than the tumor itself, as MCC tumors lack detectable amounts of VP1 [20].

Interestingly, the detection of antibodies against the viral oncoprotein small T-antigen was shown to predict MCC recurrences in newly diagnosed MCC patients [23,24].

### 3.3. MCPyV Prevalence in Non-Cutaneous Sites

The skin is a major site of viral infection, nevertheless; MCPyV has also been detected in other tissues and peripheral blood, as reviewed by Csoboz and colleagues [25].

MCPyV DNA has been found by PCR in samples from patients with upper or lower respiratory tract infections. About 2% of nasal swabs, as well as 1.3% to 4.3% of nasopharyngeal aspirate samples were MCPyV DNA-positive, but in a subset of the latter co-infections, other respiratory viruses were detected. [26,27,28]. MCPyV was found in respiratory tract samples of children aged 26 days to 7 months, as well as in immunosuppressed and immunocompetent adults [27]. In one study, MCPyV DNA was found in bronchioalveolar or bronchioaspirate samples in 17.24% of adult patients admitted to the hospital (most patients were from an intensive care unit or had hematological disorders) [29]. However, a causal role of MCPyV in respiratory disease has not been established.

Furthermore, in adults, MCPyV DNA was detected in 3.5 to 10% of non-malignant tonsils, and prevalence increased with age. Early MCPyV transcripts were found in one study [30]. MCPyV DNA detection rates were higher in tonsillar cancers (21–36%), but only low viral loads were found in both the tumor and healthy tissue [28,30,31].

Regarding blood samples, the whole-genome sequencing of blood from 8240 individuals revealed MCPyV DNA in 49 (0.6%) subjects [32]. In people with underlying immunosuppression, a higher prevalence for MCPyV DNA was observed, e.g., viral DNA was detected in the sera of 39% of HIV-1-positive patients who had not received anti-retroviral therapy versus 5.5% in HIV-negative individuals [33]. Additionally, in 22% of buffy coats from patients with chronic lymphocytic leukemia, MCPyV DNA was detected [34]. Although 2.2% of hematolymphoid malignancies were positive for MCPyV DNA, only low viral copy numbers were found, and none of the lymphoid malignancies investigated by tissue microarrays expressed the oncogenic MCPyV LT antigen in tumor cells. It has been speculated that lymphocytes may serve as a reservoir for MCPyV, but hematolymphoid malignancies associated with MCC are unlikely to be caused by MCPyV [35].

By screening non-neoplastic samples from 41 autopsy cases, MCPyV DNA could be detected in 53% of skin samples, in 27% of adrenal gland tissue, as well as in a further 16 organs (e.g., thyroid gland, spleen, bone marrow, stomach, gallbladder, pancreas, heart, and aorta). As MCPyV DNA loads were very low in all tissues, these findings need further investigation [36].

## 4. Merkel Cell Carcinoma

### 4.1. MCC Entities

**Figure 2:**
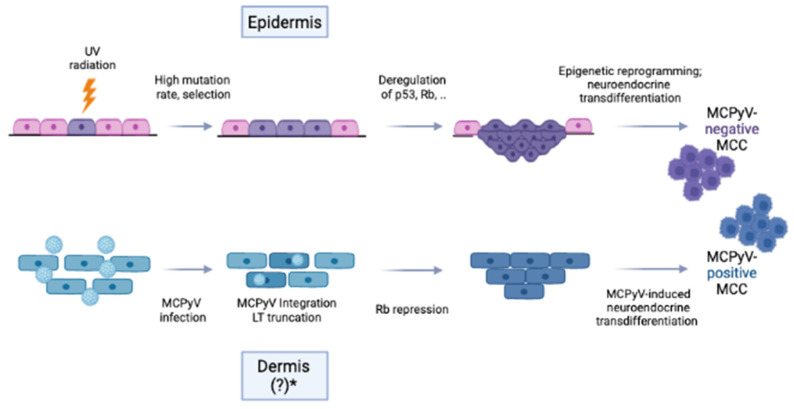
UV radiation-induced and MCPyV-induced MCC. Exposure of the skin to UV radiation (top) or integration of MCPyV DNA into the cellular genome (bottom) can induce neuroendocrine transdifferentiation and give rise to two different MCC entities. * The cell of origin for MCPyV-positive MCC is currently under debate. LT: large T-antigen; Rb: retinoblastoma protein; Figure created with BioRender.com (accessed on 5 October 2022).

MCC is a relatively rare but highly aggressive skin cancer of ectodermal origin with epithelial and neuroendocrine differentiation. Two distinct entities of MCC exist and are defined by the underlying carcinogenic mechanism: viral-induced MCC (MCPyV-positive MCC), caused by the integration of MCPyV into the host genome and viral oncogene expression or MCPyV-negative MCC caused by chronic UV radiation exposure leading to a high mutational burden of the cellular genome [37] (Figure 2).

In MCPyV-positive tumors, the clonal integration of viral DNA into the cellular genome occurs at different genome positions in different MCC and contributes to oncogenesis. It has been shown that MCPyV DNA is detectable in up to 80% of MCC [1,38,39]. with an average of 5.2 (range 0.8–14.3) T-antigen DNA copies per cell. The LT-protein is localized within the nuclei of tumor cells [1,35].

UV-driven MCC is more prevalent with closer proximity to the equator [40]. MCPyV-negative MCC have higher mutational loads with UV signatures, which are comparable to those of cutaneous squamous cell carcinoma (SCC), often affecting the RB transcriptional corepressor 1 (RB1) and/or the tumor protein p53 (TP53). Modifications in the retinoblastoma (pRb) pathway can lead to MCC development, as its disruption not only leads to the deregulation of the cell cycle but also induces neuroendocrine transformation via SOX2 expression. Similarly, in MCPyV-positive tumors, the pRb pathway is targeted by the binding of the MCPyV-encoded (truncated) large T (LT)-antigen to pRb, leading to the repression of pRB functions. Thus, in MCPyV-positive and -negative MCCs, the same key pathway is deregulated but in different pathogenetic ways [41]. Additionally, despite various genetic differences, both MCC etiologies exhibit nuclear accumulation of oncogenic transcription factors, such as the nuclear factor of activated T cells (NFAT), P-CREB, and P-STAT3, indicating commonly deregulated pathogenic mechanisms [42]. Differences in the underlying causative agent can lead, for example, to distinct morphology and localization in the body [43].

The cells of origin for both types of MCC have been a matter of debate. Despite the common name, it is unlikely that MCC derives from Merkel cells which pre-dominantly function as mechanoreceptors. The common sites of MCC do not correlate with the common locations of Merkel cells. In addition to epidermal (precursor-) Merkel cells (sensory cells localized to areas of high tactile acuity) or (pre)-B-cells, the most prominent hypothesis discusses a mesenchymal origin of MCPyV-positive MCC (dermal fibroblasts) and an epithelial origin of UV-driven MCC (epidermal keratinocytes) (Figure 2) [37]. Most MCC are developed de novo. Occasionally, MCCs are found in combination with other non-neuroendocrine carcinomas, most often UV-induced SCC, supporting the assumption of epithelial cells as the origin of MCPyV-negative MCC [44].

Doubt has arisen as to the cells of origin of MCPyV-positive MCC. It seems unlikely that a virus should be able to induce similar tumors in cells from different germ layers. From a very recent study on DNA-methylation patterns in epithelial and non-epithelial neuroendocrine cancers and MCC-derived cell lines, a common epithelial origin of both MCPyV-positive and -negative MCC cell lines has been suggested. Therefore, the observed differences may be attributed to viral vs. mutation-driven carcinogenesis rather than to the distinct cells of origin [45].

**Figure 3:**
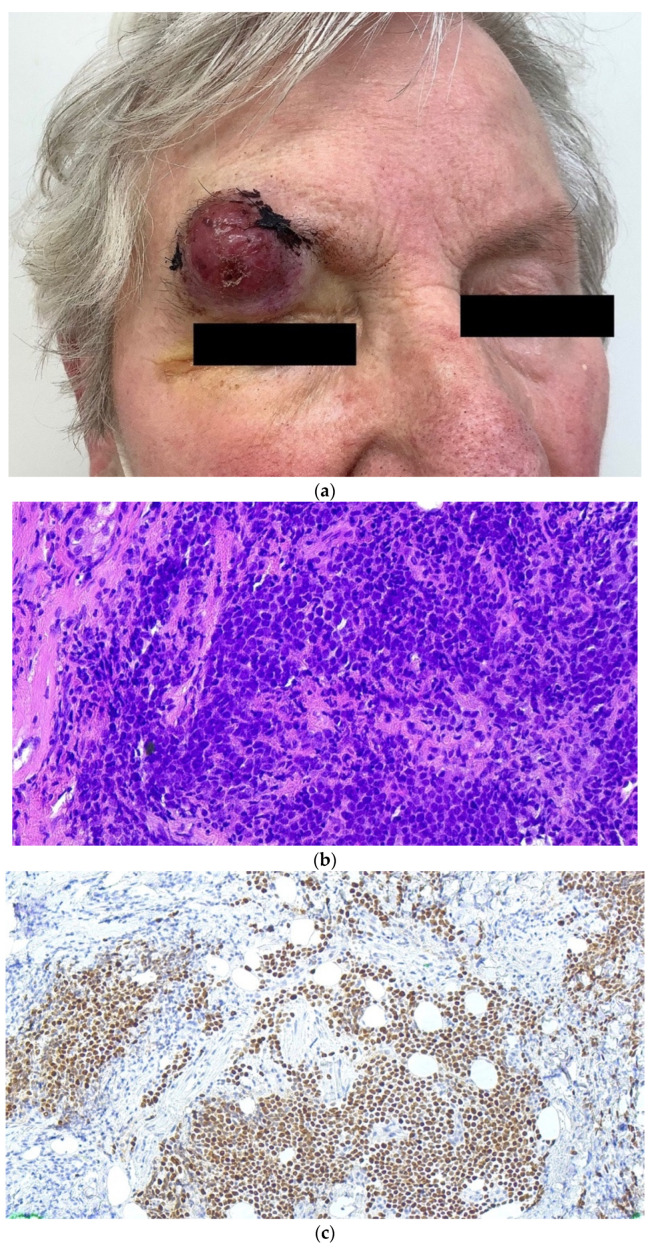
Advanced Merkel cell carcinoma in an 82-year-old patient. MCC appears as pink to reddish-livid and sometimes skin-colored, often fast-growing, and painless, shiny nodules or plaques. The main features are described by the acronym AEIOU: asymptomatic, (rapidly) expanding nodules, immunosuppressed, older than 50 years, and UV radiation exposure [50]. (**a**) Merkel cell carcinoma above the right eye covering the eyebrow. Clinically, a large, ulcerated tumor nodule, between 3 and 4 cm in diameter, with central erosions and hemorrhagic crusts is seen. According to the patient, the tumor developed within a few months. (**b**) The histology of Merkel cell carcinoma is shown in (**a**). Proliferates of small to medium-sized, hyperchromatic, densely arranged, monomorphic basophilic cells with disrupted chromatin scaffolds and mitoses are seen (Hematoxylin-eosin stain, original magnification, 100:1). (**c**) Immunohistochemical staining with the anti-MCPyV large T-antigen mouse monoclonal antibody CM2B4 (Santa Cruz Biotechnology, Inc., Santa Cruz, California, United States, 200 µg/mL). Strong expression (brown stained cells) of the large T antigen in tumor cells is visible (original magnification, 100:1).

Clinical aspects of MCC are shown in Figure 3 and are described in the legend of the figure.

Clinical suspicion of MCC should be confirmed histologically and immuno-histochemically (Figure 3b,c), as the described features are nonspecific, and often MCC lesions are presumed to be benign in nature with differential diagnosis including cyst, lipoma, or folliculitis [46]. Early diagnosis and associated smaller tumor diameter and lower tumor stages are critical for the prognosis of MCC [47,48]. The latest developments in the diagnosis and treatment of MCC have been reviewed recently in this and other journals [49,50,51,52,53,54].

### 4.2. Trends in MCC Incidence

Since the end of the last millennium, a steady increase in MCC incidence rates (new diagnoses per 100,000 per year) from 2% to 4% has been reported from different countries around the world. MCC incidence rates range from 0.1 to 2.5 cases per 100.000 individuals per year. The highest MCC incidence rates have been described in Australia (3.9 for men and 1.5 for women) and the lowest in Norway (0.45 in men, 0.22 in women) [55]. In the US, the incidence was higher in men (1.03, 61.5% of new cases) than in women (0.45, 38.5% of new cases), which has also been observed in most studies worldwide [56,57]. As an exception, a female predominance (53.8% of new cases) was observed in Ireland [58]. The incidence rates determined in different studies from countries all over the world are summarized in Table 1.

**Figure 4:**
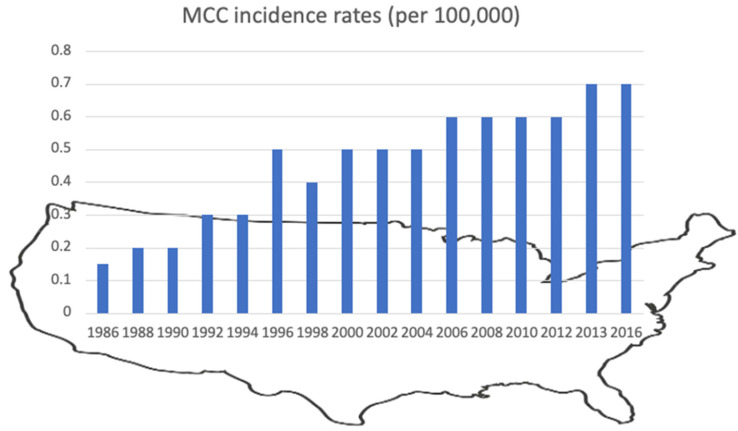
MCC incident rates per 100,000 per year in the US between 1986 and 2016 according to the surveillance, epidemiology, and end results (SEER) program (Data from [79] and SEER report 2018 [57].

In 1997, the US incidence rates for MCC reached 0.55 in men and 0.28 in women. The growing number of diagnosed MCC cases in the nineties of the last century might be attributable to increased detection rates due to improved diagnostics and enhanced attention [56,57]. Between 2000 and 2013, the number of diagnosed MCC cases nearly doubled (to 2488 cases in 2013), leading to a 95% rise in incidence (Figure 4). This rise greatly exceeded that documented for all solid cancers (15% increase) or melanoma (56% increase) in the same time period [57].

From 2006 to 2015, an average of 1972 MCC cases were diagnosed per year in the US. The relatively large number of cases allowed for the tracking of different (demographic) trends, for example, increased incidence rates with increasing age among men and women, higher incidence rates among non-Hispanic Whites, patients with localized stage, and tumors located on the head and neck [80].

In 2016, the age-standardized MCC incidence rate in the US increased to 1.03 in men and 0.45 in women. A possible reason for this could be intensified UV radiation exposure and the steadily aging population [56,57]. These projections imply that in 2025 about 3284 cases, and in 2030 approximately 5130 new cases will occur in the US [57,78].

The Surveillance of Rare Cancers in Europe (RARECARE) database reported an incidence of 0.13 per 100 000 people years between 1995 and 2002, resulting in an estimated number of 600 new MCC cases every year within the European Union [81]. The highest incidence in Europe was reported in Great Britain in a regional study between 2004 and 2013 at 1.78 per 100 000 people years. This was twelve-fold higher than the previously reported British mean [82].

In the Northern Hemisphere, the majority of MCCs is associated with MCPyV, including the US (~80%), Europe (~70–85%), and Japan (~90%), and the expression of the viral oncogenes LT- and sT-antigen in tumor cells can regularly be observed. In contrast, in Australia, the proportion of MCPyV-positive MCC is only about 20–30%, and the majority of MCC is caused by extensive UV-induced mutations [35,83]. The by far highest MCC incidence rate (26.8 per 100 000 individuals) has been reported in Australia between 2012 and 2016 in males 85 years old and over [61].

Interestingly, MCC incidence rates remained relatively stable in some populations, such as men of color from the US, men from Japan, Norway, and Denmark, and women from Denmark, Norway, and Sweden. In the period from 2003 to 2007, the incidence was highest in Australia, New Zealand, the United States, and Israel among men and in New Zealand, Australia, Ireland, and the Netherlands among women. The incidence of MCC among white non-Hispanic males in North America was positively associated with living closer to the equator. The proportion of MCC on the head was higher with advanced age [55].

The collection of comprehensive data allowed another trend to be observed. In 1986, incidence and mortality rates per 100,000 were 0.22 and 0.03 in the US, and until 2011 increased to 0.79 and 0.43, respectively. There has been a greater than 333 percent increase in mortality from MCC [77]. Similarly, in Italy, the age-adjusted mortality was 0.031/100,000, with a significant trend of increase between 1995–2006 and a slight north–south gradient [65].

### 4.3. MCC Characteristics and Risk Factors

To characterize the common features of MCC, a study from the US was conducted based on the National Cancer Institute’s SEER (Surveillance, Epidemiology, and End Results) program from 1973 to 2006, including over 3800 MCC cases [84].

**Figure 5:**
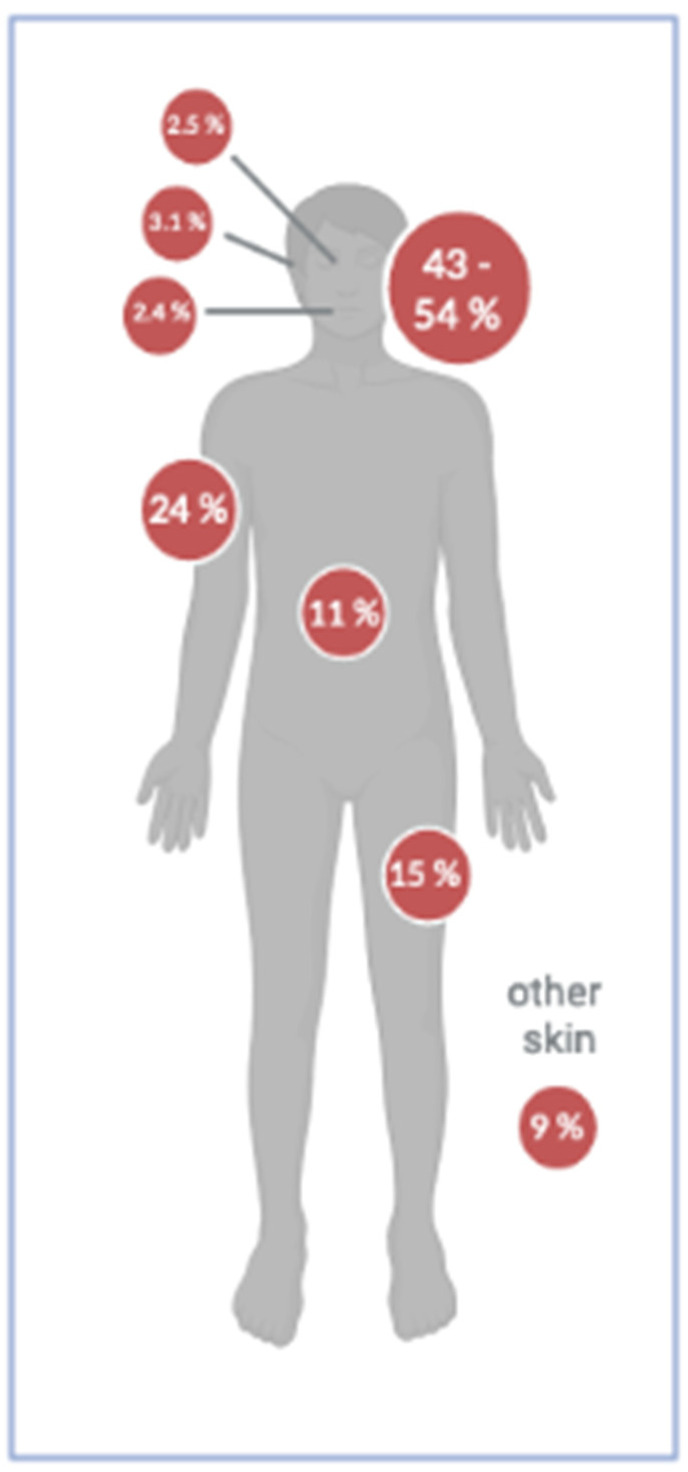
Most common body sites for MCC in percent. MCC are most commonly located on the head and neck (43–54%), followed by the upper and lower extremities (see text for references). Figure created with BioRender.com accessed on 13 December 2022.

MCC most commonly developed on the sun-exposed skin of the head and neck (43–54%), followed by upper limbs and shoulder (24%), lower limbs and hip (15%), trunk (11%), other skin (9.0%), and to a lesser extent on further localizations. An estimated 2.5–10% arise on the eyelids or periocular skin (Figure 5) [84,85]. It was observed that MCC developed slightly more frequently on the left than on the right side of the body [86].

Furthermore, most patients are fair-skinned (94.9%), and MCC is rare in people of color. Men are affected about twice as often as women [56]. At the time of MCC diagnosis, the vast majority of patients are between 60 and 85 years of age, and there is a peak in incidence in over 85-year-old individuals. In two European studies, most MCC cases were found in the age category 65+ and in people ≥70 years of age (74%) [69,81]. The median age at diagnosis was 77–77.6 years, with a slightly younger age in males (75.1 years) compared to females (79.7 years) [87]. Interestingly, a study focusing on MCC of the lower limb and hip observed a slightly younger age at first diagnosis (mean age: 72.7 years) [88].

Additionally, patients with a confirmed first primary MCC had more than twice the expected number of subsequent primary cancers. Conversely, people who were initially diagnosed with cancers other than MCC were about two and a half times more likely to have a subsequent primary MCC compared with the general population [60].

An elevated risk for the development of MCC was observed in individuals more than one year after a diagnosis of SCC of the skin (standardized incidence ratio (SIR): 14.6), basal cell carcinoma (SIR 4.3), malignant melanoma (SIR 3.3), chronic lymphocytic leukemia (CLL) (SIR 12.0–15.7), Hodgkin lymphoma (SIR 17.6), and non-Hodgkin lymphoma (SIR 4.3–5.6) [66,89,90].

An important risk factor for MCC development is immunosuppression, e.g., from organ transplantation, underlying malignant diseases, as well as treatment with immune-modulating agents. In line with this, a current, past, or concurrent diagnosis of CLL or other hematologic malignancies is more frequent in patients with MCC. Increased risk rates for MCC have been reported in patients with CLL (30-fold) or a history of organ transplantation (23.8-fold) [91].

In an Irish study on MCC incidence, 10 out of 314 MCC cases were identified to have developed in organ transplant recipients (OTR). In the normal population, the median age at diagnosis was 77.6 years. In contrast, OTR who developed MCC were diagnosed at a younger median age of 65.1 years, and the SIR for MCC (59.96) was very high [58].

People living with HIV (PLWH) have an elevated risk for various opportunistic infections and malignancies, e.g., Kaposi sarcoma, melanoma, cutaneous lymphoma, or HPV-associated tumors [92]. In an earlier review, it was stated that PLWH has a thirteen times higher risk of developing MCC. Fourteen MCC cases were analyzed, and the average age at diagnosis (49 years) was considerably lower than in the general population. Five patients developed MCC of the head and neck, whereas nine patients had lesions on other sites, which is different from the general population, where the majority of MCC is localized on the head and neck. The average duration from the time of HIV diagnosis to MCC diagnosis was 9.5 years, and the average CD4 cell count was low (256 cells/µL) [93].

In a recent study from the US during the antiretroviral therapy (ART) era (1996–2018), the SIRs in PLWH for different non-keratinocyte skin cancers (NKSCs) compared to the SIRs in the general population were determined. Elevated incidence was observed for virus-related NKSCs, namely MCC, which had a SIR of 3.15 (95%CI 1.93–4.87) predominantly on sun-exposed skin but only among PLWH with a prior AIDS diagnosis [92].

Case reports support the findings that in PLWH, the development of MCC might have unusual features, e.g., a male Caucasian with HIV primary diagnosis and a solitary nodule on the left arm above the elbow or an unusual metastasis of an MCPyV-positive MCC in the mandibular gingiva of an HIV-positive patient [94,95].

Despite improving immunity in PLWH through the use of ART, increased attention is recommended as MCC development in these patients can be faster than in the general population, occur at an earlier age, and might be in uncommon localization [96].

### 4.4. MCC Recurrence and Survival

MCC of the head and neck is highly malignant due to the early development of metastases and frequent recurrences. A recent prospective cohort study showed that MCC has a significantly higher 5-year recurrence rate (approximately 40%) than other malignancies of the skin, e.g., invasive malignant melanoma (19%) or SCC (5–9%) [48]. In a pilot study, it could be shown that similar to other malignancies, MCC changes in the hydroxymethylation pathway may be of relevance for progression [97].

In comprehensive studies from different countries, 1-year, 5-year, and 10-year relative survival was 85%, 42.4–70%, and 47%, respectively [77,98]. The wide range of 5-year survival rates might be due to different male-to-female ratios in the respective study cohorts or to differences in disease status at diagnosis. For example, 5-year survival for patients with localized and non-localized diseases were 55% and 84%, respectively [66]. The 10-year relative survival rate was significantly higher in women than in men (64.8% vs. 50.5%), and women had a significant survival advantage at 5 years compared to men (66.0% vs. 56.8%), which was due to reduced MCC-specific mortality (5-year cumulative incidence: 16.4% vs. 26.7%) [99]. Patients between 50 and 69 years of age had the highest 10-year relative survival rate (59.6%). The stage of disease was the best predictor of relative survival [84]. Predictors of decreased disease-specific survival at diagnosis were age ≥75 years, Caucasian skin type, tumor spread, and lymph node involvement. The MCC primary site had an impact on disease-specific death, and cumulative incidence of MCC-specific mortality was highest for tumors involving the scalp/neck among localized MCC (26.0%). Tumors involving the lip had the highest cumulative incidence of MCC-specific mortality among MCC with regional and distant metastasis (56.7% and 82.1%, respectively). The probability of MCC disease-specific death varies by primary site and may be a useful prognostic marker for MCC [100].

Patients with MCC of the lower limb and hip have better overall survival (68 months) than those with tumors of other skin localizations, maybe due to a lower age at first diagnosis [88]. In a very recent meta-analysis including fourteen studies with nearly 1600 patients, a significant correlation between MCPyV positivity and better overall survival (hazard ratio (HR) 0.61), as well as an improved progression-free survival (HR 0.61), was shown [101].

In a study on solid-organ transplant recipients, MCC was diagnosed at a significantly younger age in solid-organ transplant recipients compared to the matched controls (69 vs. 78 years). The MCPyV detection rate in the MCC of solid-organ transplant recipients (33%) was much lower than in the MCC of immunocompetent people (91%). In general, MCPyV-negative MCC is associated with a worse prognosis. Consequently, solid-organ transplantation was associated with an increased cumulative incidence of progression (standard hazard ratio (SHR): 3.35), MCC-specific mortality (SHR: 2.55), and overall mortality (HR: 3.26) [102]. In another study, a high proportion (70%) of OTR died from MCC, and the median survival was only 0.14 years [58].

On the molecular level, different markers linked to survival have been investigated, and the phosphorylated CRE-binding protein was found to be an independent survival factor. Immuno-expression of LRIG1 was correlated with better overall and MCC-specific survival [42,103].

### 4.5. Non-Cutaneous MCC

In an analysis of the US SEER database, which comprises 3870 MCC, 47 cases (1.2%) had a non-cutaneous localization. The lip, salivary glands, nasal cavity, lymph nodes, vulva, vagina, and esophagus were the most common extracutaneous sites [84]. In some cases, MCC exceptionally presents with only a basin lymph node localization, with neither a cutaneous primary site nor distant metastases. In a single-center series including 55 patients, inguinal lymph nodes were the most common anatomic site [104].

## 5. MCPyV in Malignancies Other than MCC

MCPyV has not only been detected in MCC but also in other skin tumors, but much less frequently than in MCC [38,105,106].

A recent meta-analysis including 50 studies with 5428 patients analyzed MCPyV in MCC, other skin lesions, and normal skin. MCPyV prevalence was much higher in MCC (80%) than in other skin lesions (4–21%) and in normal skin (11%) (odds ratio (OR) 3.51). MCPyV prevalence rates of non-MCC skin lesions included in the meta-analysis were 21% (Bowen’s disease), 20% (keratoacanthoma), 15% (squamous cell carcinoma), 15% (basal cell carcinoma), 10% (seborrheic keratosis), 6% (actinic keratosis), and 4% (melanoma) [38].

In contrast to MCC, the MCPyV large T-antigen is usually not expressed in these tumors, and the viral load is much lower than that found in MCC, arguing against a causal role of MCPyV in skin tumors other than MCC [105,106].

The role of MCPyV has been discussed in primary cutaneous B- and T-cell lymphomas, especially in folliculotropic mycosis fungoides (fMF). MCPyV DNA was found in 50–75% of fMF samples, with higher viral loads than in healthy skin, T-cell mediated skin benign infiltrates, or other types of cutaneous lymphomas [107,108]. Large T antigen expression, however, could only be demonstrated in two of the 24 fMF analyzed in one study [108] and none of the eight fMF investigated in another study [107].

Studies displaying the presence of the virus in various non-MCC tumors were not able to clearly demonstrate a direct connection between cellular transformation and the presence of the virus [109]. The prevalence of MCPyV is significantly lower in non-MCC tumors compared to MCC, with lower viral loads and sparse viral protein expression. Moreover, the state of the viral genome and whether a truncated large T antigen is expressed has rarely been investigated [25].

## 6. Summary/Conclusions

MCC is a rare but highly malignant neuroendocrine tumor of the skin whose incidence is increasing worldwide. Two MCC entities have been described to be caused by different etiological factors (MCPyV or UV radiation). Although MCPyV is a commensal virus of the human skin, poorly controlled MCPyV infection with high viral loads due to an aging (immunosenescence) or suppressed immune system and/or chronically UV-damaged skin may pave the way to MCPyV-associated MCC. About 80% of the tumors are associated with MCPyV, and integration of the virus into the host genome drives tumorigenesis. Individuals of the male sex, older age (>75 years), and fair skin type (particularly those with a history of prolonged sun exposure) are most susceptible to the disease. Chronic T cell immunosuppression is a key feature in MCC development, and in patients with chronic lymphocytic leukemia, Hodgkin lymphoma, and other malignancies, a history of organ transplantation or HIV infection significantly clusters in cohorts of MCC cases. Hypotheses to explain the global increase in MCC incidence include more refined diagnostic strategies, improved awareness, intensified data collection, and scientific research, as well as increased life spans, more immunosuppressed patients, and enhanced UV exposure in many countries.

## Figures and Tables

**Table 1:** Worldwide MCC incidence rates. CI = confidence interval; y = year; **bold** = age-adjusted and gender-specific incidence rates in the respective year (annual percentage over surveillance period given); * per 100,000 people years; ** in men 66–85 years old between 2012 and 2016; *** European- and world-standardized incidence rate; ^§^ increase over time period; ° per 100 000 standardized to the US standard 2000 population; °° crude incidence.

Country/Region	Age-Adjusted Incidence Rate *	Annual Percentage Increase (CI 95%)	Gender-Specific Incidence Rates *(CI 95%)		MCC Cases Included (n)	Surveillance Period	References
			men	women			
Australia (Western)			1.0	0.63	215	1993–2007	[59]
Australia (Queensland)	1.6	2.6%	2.5	0.9	340	2006–2010	[60]
Australia (Queensland)		1.8% (0.7–2.8)	2.99	0.90		1997–**2016**	[56]
Australia (Victoria)	2.5	4.2% (2.8–5.8) **	3.9	1.5	1095	1986–2016	[61]
New Zealand	0.88		1.05	0.74	356	2002–2011	[62]
New Zealand	0.96 ° (0.88–1.04)		1.45 times that of females (1.23–1.7)		601	2000–2015	[63]
New Zealand		2.0% (0.4–3.7)	1.37	0.95		1997–**2016**	[56]
Europe	0.13 °°					1995–2002	[64,65]
Denmark	0.22		0.2	0.25	185	1995–2006	[66]
Finland			0.11	0.12	181	1989–2008	[67]
France			0.26 and 0.43 ***	0.24 and 0.38 ***	290	2006–2010	[68]
France (Bas-Rhin)	0.23 (0.13–0.33)	5.14% (2.31–8.34%)	0.18 (0.13–0.33)	0.16 (0.12–0.21)	111	2010–2013	[69]
Germany			0.40	0.30	1848	1988–2010	[70]
Ireland	0.41 (0.31–0.51)		0.58 (0.5–0.75)	0.28 (0.18–0.38)	314	2009–2014	[58]
Italy	0.28 (0.25–0.32)					2001–2005	[65]
Netherlands	0.17/0.35				808	1993–1997/2003–2007	[71]
Norway		4.0% (2.1–5.9)	0.45	0.22		1997–**2016**	[56]
Great Britain (Scotland)		3.7% (2.0–5.5)	0.50	0.44		1997–**2016**	[56]
Great Britain (Southeast-Scotland)	0.133				20	1993–2003	[72]
Great Britain (England)	0.10–0.20					1999–2008	[64]
Great Britain (East-England)	0.21		0.19	0.24	73	2004–2013	[73]
Spain	0.11 °°				15	1994–2004	[74]
Sweden	0.45 (age ≥ 85 years)		0.42 (0.41–0.43)	0.33 (0.32–0.33)		1990–2005	[75]
Sweden	0.11 to 0.19	73% ^§^	0.09 to 0.20	0.12 to 0.17		1993–2012	[76]
United States	0.6				1500/y	2009	[64]
United States	0.79					2011	[77]
United States	0.7 (0.7–0.8)					2013	[57]
United States		2.7% (2.0–3.3)	1.03	0.45	3063	1997–**2016**	[56]
US Non-Hispanic Whites		3.0% (2.4–3.7)	1.22	0.54	3063	1997–**2016**	[56]
United States	0.66 (0.62–0.70)				3720	2012–2016	[78]
